# Analysis of Structure and Dynamics in Three-Neuron Motifs

**DOI:** 10.3389/fncom.2019.00005

**Published:** 2019-02-07

**Authors:** Patrick Krauss, Alexandra Zankl, Achim Schilling, Holger Schulze, Claus Metzner

**Affiliations:** ^1^Experimental Otolaryngology, Neuroscience Lab, University Hospital Erlangen, Friedrich-Alexander University Erlangen-Nürnberg (FAU), Erlangen, Germany; ^2^Department of Physics, Chair for Biophysics, Friedrich-Alexander University Erlangen-Nürnberg (FAU), Erlangen, Germany

**Keywords:** three-node network motifs, neural networks, Boltzmann neurons, structure, dynamics

## Abstract

Recurrent neural networks can produce ongoing state-to-state transitions without any driving inputs, and the dynamical properties of these transitions are determined by the neuronal connection strengths. Due to non-linearity, it is not clear how strongly the system dynamics is affected by discrete local changes in the connection structure, such as the removal, addition, or sign-switching of individual connections. Moreover, there are no suitable metrics to quantify structural and dynamical differences between two given networks with arbitrarily indexed neurons. In this work, we present such permutation-invariant metrics and apply them to motifs of three binary neurons with discrete ternary connection strengths, an important class of building blocks in biological networks. Using multidimensional scaling, we then study the similarity relations between all 3,411 topologically distinct motifs with regard to structure and dynamics, revealing a strong clustering and various symmetries. As expected, the structural and dynamical distance between pairs of motifs show a significant positive correlation. Strikingly, however, the key parameter controlling motif dynamics turns out to be the ratio of excitatory to inhibitory connections.

## Introduction

Recently, a number of projects seek to map the human connectome, aiming to connect its structure to function and behavior (Markram, 2012; Van Essen et al., 2013; Glasser et al., 2016). However, even if the connectome would be known completely, it remains an unresolved problem how to translate this detailed structural data into meaningful information processing functions and algorithms (Jonas and Kording, 2017). For instance, the connectome of *C. elegans* has been known for decades, and involves only 302 neurons. Nevertheless, even this relatively small system is not yet understood in terms of its dynamics, let alone at a functional level (Hobert, 2003; Gray et al., 2005).

Moreover, the problem is complicated by the fact that very similar dynamics of a neural network at a macroscopic level might be realized by very different structures at the microscopic level (Newman, 2003). Therefore, an important step toward extracting function from structure is a tool to quantitatively compare different structures and dynamics.

In a neural network, all relevant structural information is encoded in a weight matrix, containing the mutual connection strength of all neurons (Hertz et al., 1991; LeCun et al., 2015; Schmidhuber, 2015; Goodfellow et al., 2016). Quantifying the similarity of two weight matrices by standard measures, such as the sum of squared differences between corresponding matrix elements, is however not sufficient because of possible permutations of the neuron indices. Similarly, the dynamical properties of a neural network are encoded in a matrix of transition probabilities between all possible network states. As mentioned before, comparing the sum of squared differences between corresponding matrix elements fails in case of neuron permutations.

To solve this problem, we develop permutation-invariant metrics for the structural distance *d*_*str*_(*A, B*) and for the dynamical distance *d*_*dyn*_(*A, B*) of two given networks *A* and *B*. By construction, these distance-measures yield *d*_*str*_(*A, B*) = 0 and *d*_*dyn*_(*A, B*) = 0 whenever *B* is topologically identical to *A*, even though the corresponding weight and transition matrices of *A* and *B* may differ due to inconsistent neuron indices.

We apply these distance metrics to so-called motifs, a class of small recurrent networks which have been shown to be fundamental building blocks of various complex networks (Milo et al., 2002), such as gene regulatory networks (Shen-Orr et al., 2002; Alon, 2007), the world wide web (Milo et al., 2002), and the human brain (Song et al., 2005).

We exhaustively compute the structural and dynamical distances between all possible pairs of the 3,411 different classes of three-neuron motifs with ternary connection strengths, resulting in two distance matrices with 3,411 × 3,411 entries each. Based on these matrices, we use classical multidimensional scaling (Kruskal, 1964a,b; Cox and Cox, 2000; Borg et al., 2017; Krauss et al., 2018) to visualize the structural and dynamical similarity relations between different motifs on a two-dimensional plane.

Remarkably, it turns out that the distribution of motifs, both in structural and dynamical “space,” is not uniform, but strongly clustered and highly symmetrical. Moreover, the position of a motif within structural and dynamical space correlates with the ratio of excitatory and inhibitory connections (balance) in the motif's connection matrix.

## Methods

### Three-Neuron Motifs

Our study is based on Boltzmann neurons (Hinton and Sejnowski, 1983) without bias. The total input *z*_*i*_(*t*) of neuron *i* at time *t* is calculated as:

(1) $$\begin{array}{r} z_{i}\left(t\right)=\overset{N}{\underset{j=1}{\sum }}w_{ij}y_{j}\left(t-1\right) \end{array}$$

where *y*_*j*_(*t* − 1) is the binary state of neuron *j* at time *t* − 1 and *w*_*ij*_ is the corresponding weight from neuron *j* to neuron *i*. The probability *p*_*i*_(*t*) of neuron *i* to be in state *y*_*i*_(*t*) = 1 is given by:

(2) $$\begin{array}{r} p_{i}\left(t\right)=\sigma \left(z_{i}\left(t\right)\right), \end{array}$$

where σ(*x*) is the logistic function

(3) $$\begin{array}{r} \sigma \left(x\right)=\frac{1}{1+e^{-x}}. \end{array}$$

**Figure 1:**
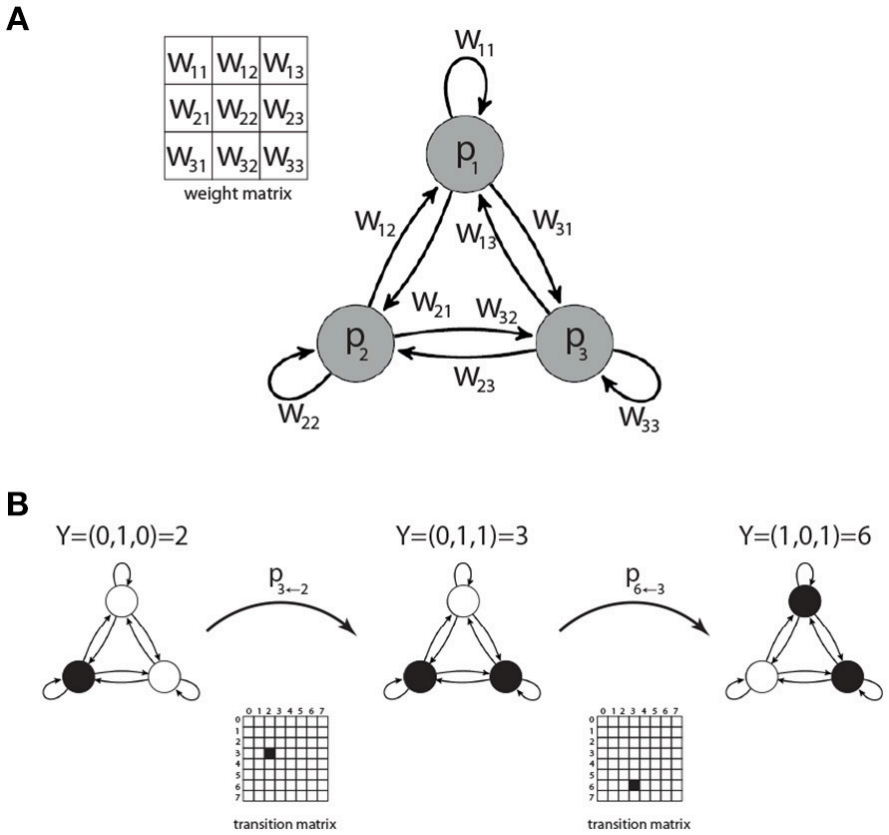
Motifs of three coupled Boltzmann neurons. Each motif is characterized by a 3 × 3 weight matrix *W* **(A)**, defining the connection strength between the neurons. There are 2^3^ = 8 possible states *Y* = 0…7 for each motif. The transition probabilities between these states are summarized in a 8 × 8 state transition matrix **(B)**.

We investigate the set of all possible network motifs that can be built from 3 Boltzmann neurons with ternary connections *w*_*ij*_ ∈ {−1, 0, +1}, where self connections *w*_*ii*_ are permitted (Figure 1A). In principle there are 3^9^ = 19, 683 possible ternary 3 × 3 weight matrices. However, due to permutation of the neuron indices, not every matrix corresponds to a unique motif class.

We have exhaustively listed all possible ternary weight matrices in a set. We then partitioned this set into equivalence classes, defining two matrices as equivalent if they can be made element-wise identical by a suitable permutation of neuron indices. By this way, we found that there are exactly 3,411 distinct motif classes. For later convenience we label all motif classes with unique indices, which are derived from the corresponding weight matrices.

### State Transition Matrices of Motifs

Since every neuron can be in one of two binary states, a 3-node motif can be in 2^3^ = 8 possible motif states. Given the momentary motif state and the weight matrix, the probabilities for all eight successive motif states can be computed, thus defining the 8 × 8 state transition matrix of a Markov process (Figure 1B). All information theoretical properties of 3-neuron motifs, such as entropy or mutual information of successive states, are determined by the state transition matrix. We therefore calculate the transition matrices for each of the 3,411 motif classes.

### Motif Classes

**Figure 2:**
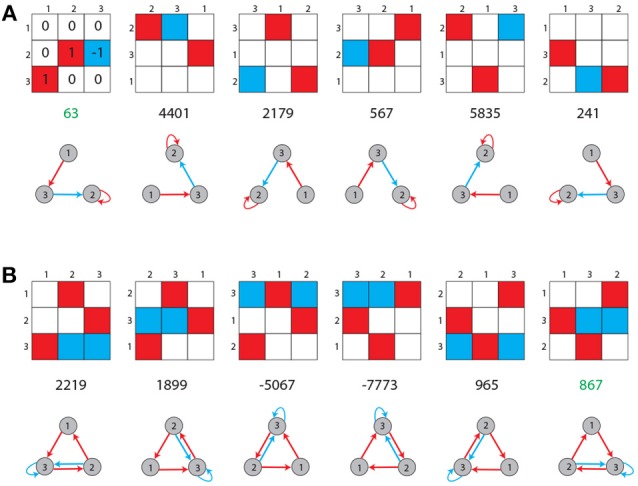
Unique labeling of motif classes. Possible entries in the 3 × 3 weight matrix of a motif are −1 (blue), 0 (white), and +1 (red). Shown are all possible permutations of topologically equivalent motifs for two arbitrary chosen cases **(A,B)**. Each motif class is assigned a unique label (green numbers), as described in the Methods section.

A motif class *A* is defined as the set {*A*^(*m*)^:*m* = 1…6} of weight matrices, which are all related to each other by index permutations, such as $a_{i,j}\to a_{i,j}^{\left(m\right)}=a_{\pi_{m}\left(i\right),\pi_{m}\left(j\right)}$, where π_*m*_ is the m-th permutation (Figure 2).

### Unique Labels of Motif Classes

The nine entries of the weight matrix $W=\left(\begin{array}{lll} a & b & c\\ d & e & f\\ g & h & i \end{array}\right)$ of one motif class are treated as a vector **w** = (*abcdefghi*). The components of this vector are then treated as the digits of a number in the ternary system:

(4) $$\begin{array}{r} a\cdot 3^{8}+b\cdot 3^{7}+c\cdot 3^{6}+d\cdot 3^{5}+e\cdot 3^{4}+f\cdot 3^{3}+g\cdot 3^{2}+h\cdot 3^{1}+i\cdot 3^{0}. \end{array}$$

It can be simplified to

(5) $$name=\sum_{i=0}^{8}w[i]\cdot 3^{8-i}.$$

Here, **w**[0] equals the first entry of the vector **w** and the value of the sum is the name of the motif. Due to the possible entries **w**[*i*] ∈ {−1, 0, 1} the motif names range between “−9,841” and “9,841,” starting with the motif with just “−1” as entries and finishing in the motif with just “1” as entries. Of course not every number in this range is assigned a motif class as there are in total only 3411 motif classes. This version of the formula is used because the motif class with just zeros as entries gets the name “0” and the names are approximately symmetrical around that motif class. Furthermore, in order to make the system more balanced, each motif class is represented by the weight matrix with the smallest absolute value of *name* among all of its permutations (Figure 2).

### Structural Distance Between Motif Classes

**Figure 3:**
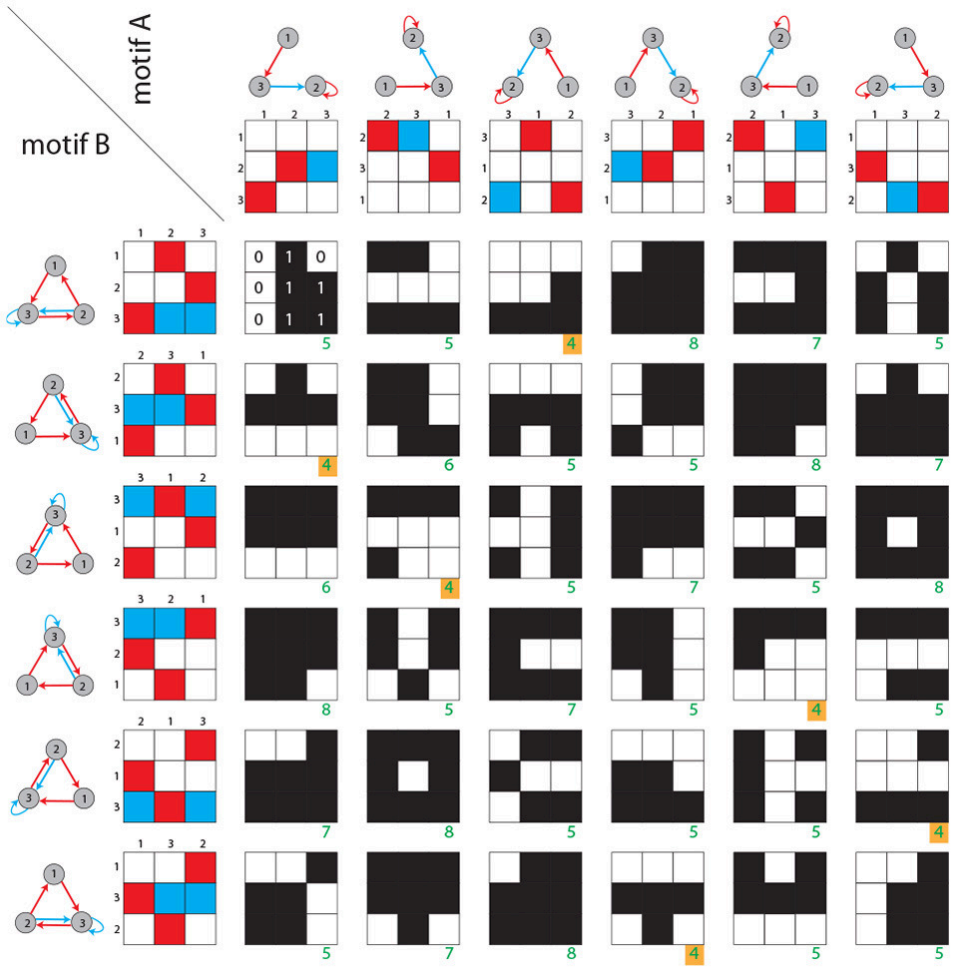
Structural distance between motif classes. Rows and columns show the six possible permutations of two given motif classes. For each of the 36 combinations, the generalized Hamming distance ĥ (green numbers) is computed. Black and white matrices indicate the Hamming distances between corresponding matrix elements. As described in the Methods section, the structural distance is defined as the minimum of all 36 generalized Hamming distances (green numbers with yellow background).

The dynamical distance is calculated as follows (Figure 3): Given are two motif classes *A, B*. For each class we derive all six permuted weight matrices *A*^(*m*)^ and *B*^(*n*)^. For each of the 36 pairs of weight matrices *A*^(*m*)^ and *B*^(*n*)^, we compute a generalized Hamming distance ĥ, defined as the number of different ternary matrix elements:

(6) $$\begin{array}{r} ĥ\left(A^{\left(m\right)},B^{\left(n\right)}\right)=\underset{i,j}{\sum }\left(1-\delta_{a_{i,j}^{\left(m\right)},b_{i,j}^{\left(n\right)}}\right), \end{array}$$

where δ_*x, y*_ is the Kronecker symbol. The structural distance *d*_*str*_ between motif classes matrices *A, B* is defined as the smallest of the above 36 Hamming distances

(7) $$\begin{array}{r} d_{str}\left(A,B\right)={\text{min}}_{m,n}\left(ĥ\left(A^{\left(m\right)},B^{\left(n\right)}\right)\right) \end{array}$$

### Dynamical Distance Between Motif Classes

**Figure 4:**
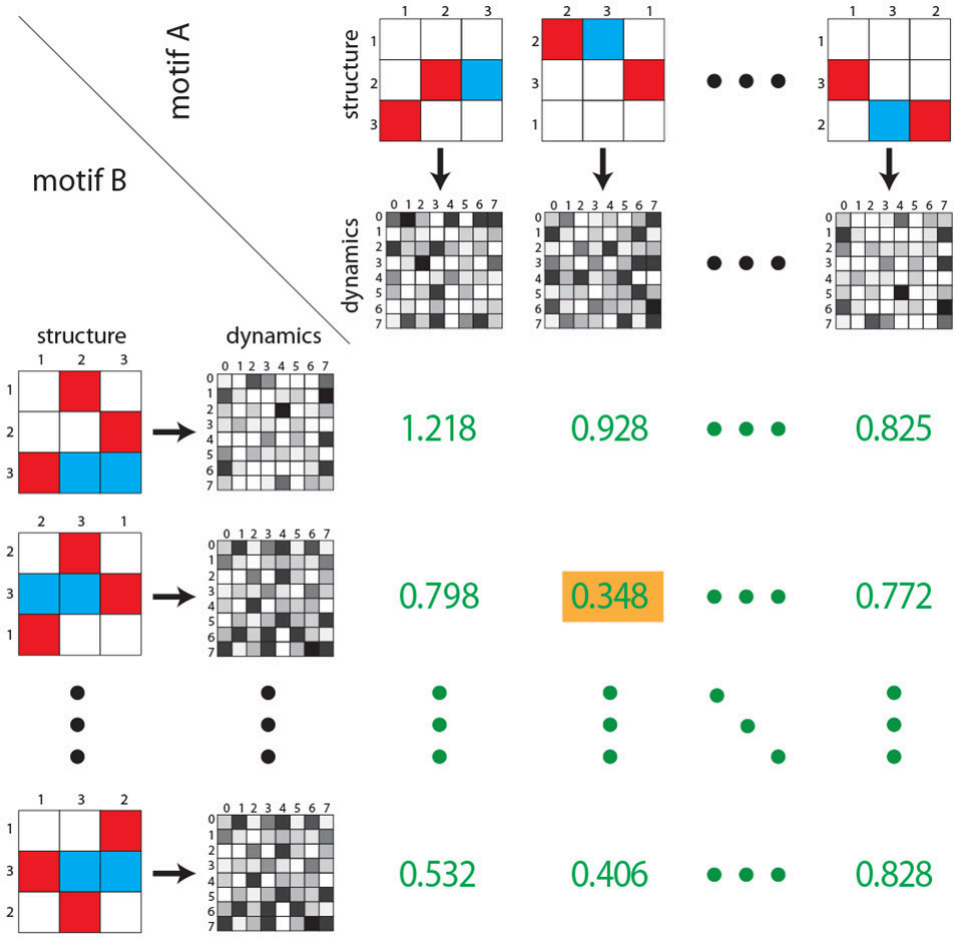
Dynamical distance between motif classes. As in Figure 3, rows and columns contain the six possible permutations of two given motif classes (structure). For each permutation the corresponding state transition matrix is calculated (gray shaded matrices). Subsequently, for each of the 36 combinations, the Euclidean distance between each pair of state transition matrices is calculated (green numbers). As described in the Methods section, the dynamical distance is defined as the minimum of all 36 Euclidean distances (green number with yellow background). Note that green numbers do not correspond to actual distances, but are for illustration purposes only.

The dynamical distance is calculated as follows (Figure 4): For each motif classes *A*, we compute general features *F*(*A*), which can be scalars, vectors or matrices. In the case of matrix-like features *F* and *G* (e.g., state transition probability matrices), the Euclidean distance is defined as

(8) $$\begin{array}{r} d\left(F,G\right)=\sqrt{\underset{i,j}{\sum }{\left(f_{i,j}-g_{i,j}\right)}^{2}} \end{array}$$

To compute the dynamical distance *d*_*dyn*_(*A, B*) between two motif classes *A* and *B*, we derive all 36 pairs of features (e.g., the state transition matrix) from permuted weight matrices (*F*(*A*^(*m*)^), *F*(*B*^(*n*)^)) and calculate the Euclidean distance *d*(*F*(*A*^(*m*)^), *F*(*B*^(*n*)^)) of each pair. The dynamical distance *d*_*dyn*_ between motif classes *A, B* is defined as the smallest of the 36 Euclidean distances

(9) $$\begin{array}{r} d_{dyn}\left(A,B\right)={\text{min}}_{m,n}\left(d\left(F\left(A^{\left(m\right)}\right),F\left(B^{\left(n\right)}\right)\right)\right) \end{array}$$

### Multidimensional Scaling

We compute the pair-wise structural and dynamical distances between all 3,411 motif classes. In order to visualize their similarity relations, we use classical multidimensional scaling (Kruskal, 1964a,b; Cox and Cox, 2000; Borg et al., 2017; Krauss et al., 2018). This method assigns to each motif class a point on the two-dimensional plane, so that the mutual geometric distances between the points reflect the structural or dynamical distances between the motif classes. In contrast to alternative visualization methods such as t-SNE (Maaten and Hinton, 2008) where the results depend crucially on the choice of parameters (Wattenberg et al., 2016), classical multidimensional scaling has no adjustable parameters and therefore produces more robust and reproducible results.

## Results

By an exhaustive listing of all possible weight matrices and a subsequent numerical sorting into equivalence classes, we could show that there exist 3,411 structurally distinct three-neuron motif classes with ternary connection strengths. We computed the structural and dynamical distances between all possible pairs of these motif classes, resulting in two 3,411 × 3,411 distance matrices.

**Figure 5:**
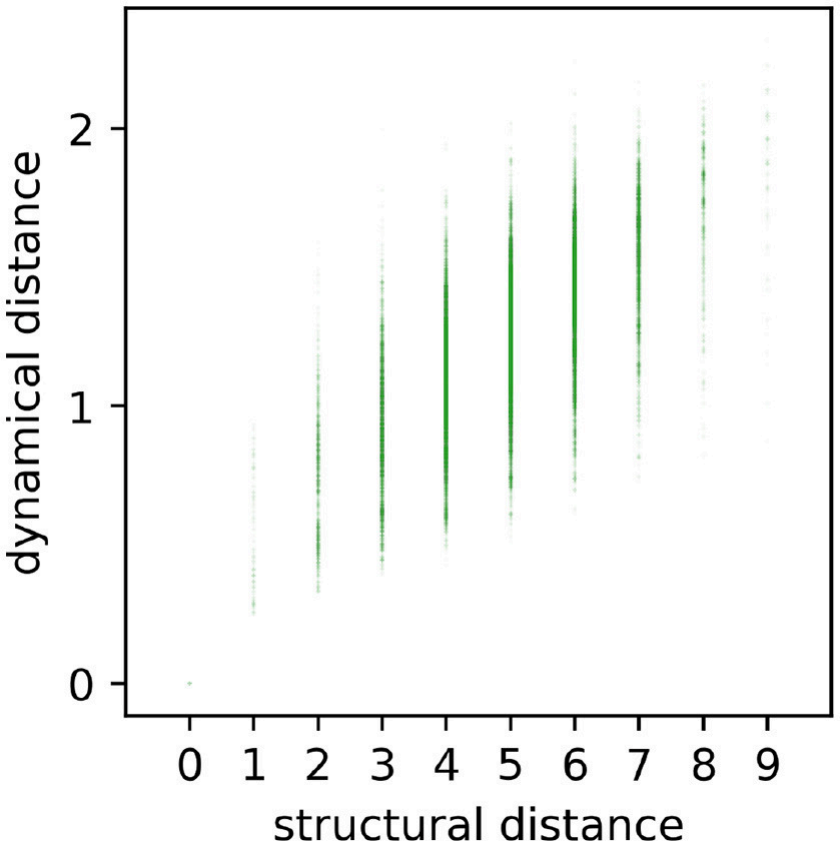
Scatterplot of all pairwise dynamical and structural distances. Each point (*d*_*str*_, *d*_*dyn*_) represents the relation between structural distance *d*_*str*_ and dynamical distance *d*_*dyn*_ of a certain motif. Dynamical and structural distances are significantly correlated (*r* = 0.59, *p* < 0.001), but also show a large variance.

In a first step, we tested the intuitive expectation that the dynamical distance *d*_*dyn*_ between motifs should grow, at least as a general trend, with their structural distance *d*_*str*_. For this purpose, we produced a scatter plot of *d*_*dyn*_ versus *d*_*str*_, including all 3, 411^2^ pairs of motif classes (Figure 5). We found that for each given structural distance (except for *d*_*str*_ = 0), the distribution of possible dynamical distances is very large. Nevertheless, there is a clear positive correlation of *r* = 0.59 (*p* < 0.001) between structure and dynamics, thus confirming the expectation.

In a next step, we investigated the similarity relations between motif classes, as they are contained in the two 3,411 × 3,411 matrices of structural and dynamical distances. For this purpose, we have used classical multidimensional scaling (MDS) (Kruskal, 1964a,b; Cox and Cox, 2000; Borg et al., 2017; Krauss et al., 2018) to arrange all motif classes as points on a two-dimensional plane, so that the mutual geometric distances between the points reflect the corresponding structural or dynamical distances.

**Figure 6:**
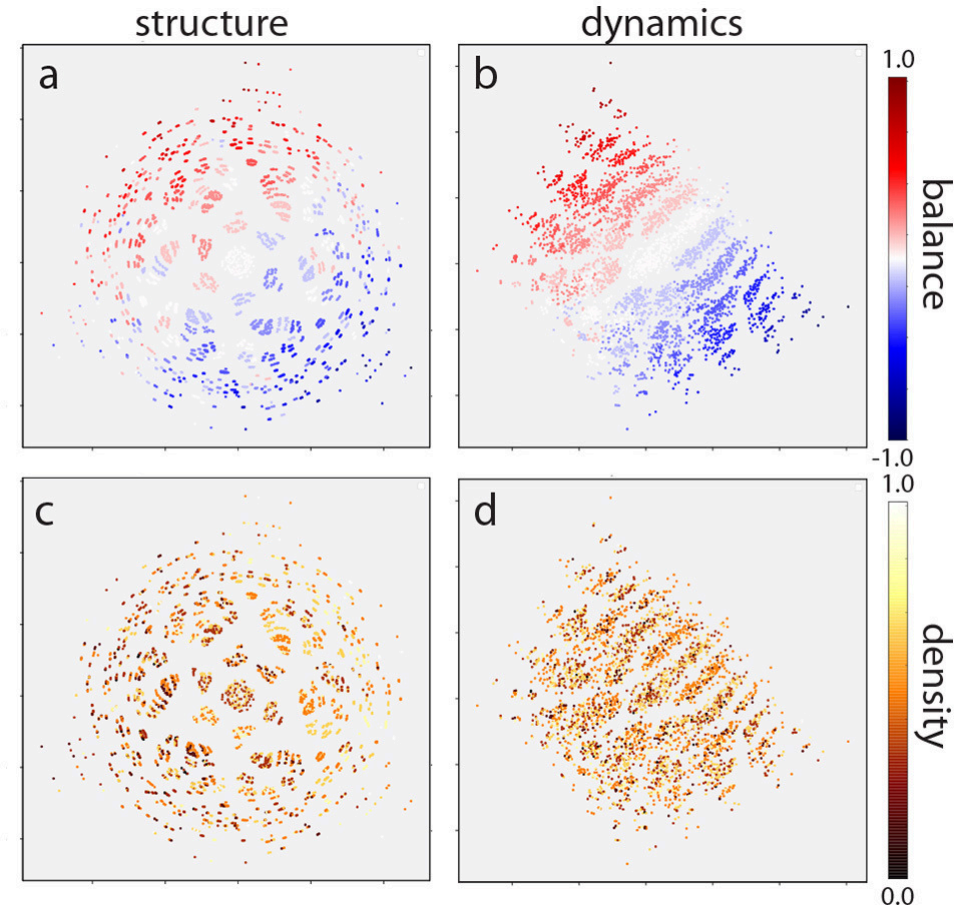
Multidimensional scaling of motif distribution in structural **(a,c)** and dynamical **(b,d)** space. Plots are color coded according to balance **(a,b)** and density **(c,d)** parameters. The structural distribution reveals a six-fold rotation symmetry due to the six possible permutations of 3-neuron motifs. In addition, motifs are ordered linearly according to the balance parameter, in both structural **(a)** and dynamical **(b)** space. By contrast, motifs are not ordered with respect to the density parameter **(c,d)**. Note that absolute coordinates of points have no particular meaning other than scaling relative distances between any pair of points.

This two-dimensional representation reveals that the distribution of motif classes in both structural and dynamical “space” is not uniform, but instead is strongly clustered (Figure 6). The structural distribution (Figures 6a,c) also reveals a six-fold rotation symmetry, which might be due to the six possible permutations of 3-neuron motifs.

As a final step, we investigated how motifs are affected by the *statistical* properties of the weight matrix. In particular, we considered the statistical parameters “density,” defined as the fraction of non-zero connections among all possible connections, as well as “balance,” the ratio between excitatory and inhibitory connections. We computed the values of these two statistical parameters for all motif classes and color-coded them correspondingly in the two-dimensional MDS representations (Figure 6).

We find that the density parameter is not at all related to the position of a motif class in the structural or dynamical plane (Figures 6c,d). By contrast, there is a clear linear ordering of motif classes with respect to the balance parameter (Figures 6a,b), both in the structural and in the dynamical plane. Indeed, altering the ratio between excitatory and inhibitory connections has a much more pronounced effect on the motif dynamics than changing the structural distance itself.

## Discussion

The relation of structure and function is a long-standing topic in biology (Bullock and Horridge, 1965; Estes and Cohen, 1989; Blackburn, 1991; Harris, 1996; Missale et al., 1998; Mitchell et al., 2011). On the one hand, the micro-structure of a biological system determines the set of possible functions that this system can serve. On the other hand, human observers may not be able to deduce the function of a system from its structure alone: even if we know all neural connection strengths in some sub-network of the animal brain, as well as all its input and output signals, the specific purpose of this sub-network within the whole of the organism may remain elusive (Hobert, 2003; Gray et al., 2005; Jonas and Kording, 2017). Indeed, “function” is not a property of the isolated subsystem alone, but can only be defined in the context of its embedding global system. For this reason, we focus in this work not on the function of neural systems, but on their dynamics—a property that is completely determined by the network structure and, if present, the system's input signals.

An additional advantage of this approach is that dynamics, just as structure, can be conveniently expressed in the form of matrices. Based on these matrices, we have developed suitable metrics that measure the distance of two neural networks in structural or dynamical space respectively. Using this tool, we can investigate how sensitive network dynamics reacts to small changes in network structure. Robustness with respect to structural changes is crucial in biological brains, as the synaptic weights cannot be adjusted with extremely high accuracy (Pinneo, 1966; Faisal et al., 2008; Rolls and Deco, 2010).

For the case of isolated three-neuron networks, we have found that the question of robustness has no definitive answer on the microscopic level of individual neuron connection strength: a small topological change in the connection matrix (i.e., adding or removing a connection, or inverting its sign), can have, both, small and large dynamical consequences. By contrast, a much clearer correlation is found between certain statistical (macroscopic) properties of a network's weight matrix and its dynamics. In particular, the ratio of excitatory to inhibitory connections (balance) affects network dynamics very strongly, while the ratio of non-zero connections (density) is much less important. This is in line with recent micro-anatomical studies of the hippocampus and the neocortex, where it was found that the balance is conserved (Megías et al., 2001; Gal et al., 2017).

This result suggests that a recurrent neural network can gain or lose a large random fraction of neural connections without drastically changing its dynamical state, provided the balance remains unchanged. We speculate that, in the brain, this surprising robustness may help to keep the cortex functional in periods of increasing density during development and contribute to the phenomenon of graceful degradation (Rolls and Treves, 1990).

In this work we abstracted from biological detail in that we included all possible three-neuron motifs with ternary connection strengths. By contrast, in the human brain the vast majority of neurons is either purely excitatory or purely inhibitory. However, there are prominent exceptions to this rule, such as the dopaminergic transmission within the basal ganglia (Kandel et al., 2000).

Future work will need to investigate whether our results extend to larger neural networks, to networks with continuous rather than ternary connection strengths between the neurons, and to networks based on alternative neuron models, such as non-probabilistic threshold units. It might also be interesting to consider networks built from mixed neuron types. Finally, we note that our choice of probabilistic Boltzmann neurons together with zero bias leads to a firing probability of 0.5 without any input, which is not biologically realistic. Neurons with a low spontaneous firing rate might lead to other interesting dynamics and might therefore also be investigated in future work.

## Author Contributions

PK and CM designed the study and developed the theoretical approach. AZ performed computer simulations. PK, AS, and AZ prepared the figures. PK and CM wrote the paper. HS and AZ provided helpful discussion. All authors read and approved the final manuscript.

### Conflict of Interest Statement

The authors declare that the research was conducted in the absence of any commercial or financial relationships that could be construed as a potential conflict of interest.
